# Dermoscopic Features of Spitz Tumor With *LMNA-NTRK1* Fusion

**DOI:** 10.5826/dpc.1101a101

**Published:** 2020-12-10

**Authors:** Ben J. Friedman, Gabrielle Robinson, Laurie Kohen

**Affiliations:** 1 Department of Dermatology, Henry Ford Health System, Detroit, MI, USA; 2 Department of Pathology and Laboratory Medicine, Henry Ford Health System, Detroit, MI, USA

**Keywords:** Dermoscopy, Spitz tumor, NTRK1 fusion, Spitz nevus

## Introduction

Spitz tumors are a heterogenous group of melanocytic neoplasms, which on histology demonstrate enlarged oval, epithelioid or spindled melanocytes, often in association with epidermal hyperplasia. The degree of pigmentation and presence or absence of maturation is variable. The molecular-genetic basis for these variations in histomorphology is increasingly being recognized. Spitz tumors typically harbor *HRAS* activating mutations, inactivation or deletion of *BAP1* or gene fusions involving the kinases *ROS1, ALK, NTRK1*, *BRAF, RET, MET* or *NTRK3* [1]. Some of these aberrations have been associated with progression to melanoma, while others have not [2].

There is little in the literature regarding the impact of particular molecular subtypes of Spitz on clinical-dermoscopic appearance. Most publications describe the clinical-dermoscopic features common to Spitz tumors as an entire category. We deduce that, as many of these neoplasms have characteristic histomorphology, clinical-dermoscopic features may be relatively consistent for a particular molecular-genetic aberration. Herein, we describe the clinical-dermoscopic features of a Spitz tumor with an *LMNA-NTRK1* fusion presenting in an adult.

## Case Presentation

A 51-year-old man with a history of numerous clinically atypical pigmented lesions, multiple biopsy-confirmed dysplastic nevi, and a first-degree relative with metastatic melanoma presented for a follow-up comprehensive skin examination. Due to the patient’s high risk, his skin surface was being surveyed longitudinally with digital whole-body photography and high-resolution dermoscopy. A new lesion was identified on his left calf, which was not present on exam 6 months prior.

**Figure 1:**
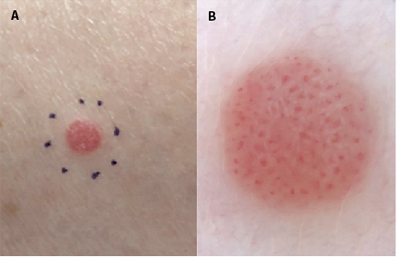
(A) Clinical: Red dome-shaped papule on left calf. (B) Dermoscopic: Regular array of glomeruloid vessels admixed with crystalline structures.

**Figure 2:**
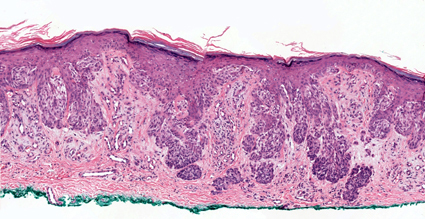
Histopathology, low power. Compound proliferation of enlarged spindled and epithelioid melanocytes with nests containing peripheral clefts. There are “filagree-like” rete ridges with lobulated nesting pattern (H&E, original magnification ×100).

**Figure 3:**
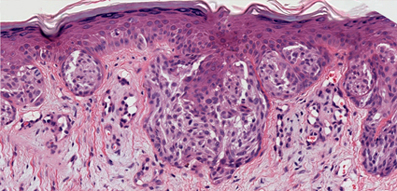
Histopathology, high power. Hypervascular papillary dermis is seen in between the altered rete pegs containing coils of dilated capillaries, which correlates to the dermoscopic vascular morphology (H&E, original magnification ×300).

Clinical examination revealed a smooth, red, dome-shaped 5 × 5 mm papule with little surface change (Figure 1A). On dermoscopy, the lesion was symmetric and demonstrated a regular array of coiled-glomerular vessels admixed with crystalline structures (Figure 1B). On histopathology, there was a broad compound proliferation of enlarged spindled and epithelioid (amelanotic) melanocytes with associated epidermal hyperplasia (Figures 2 and 3). Many junctional melanocytic nests demonstrating peripheral clefting and occasional Kamino bodies were observed. Thin and elongated “filigree-like” rete ridges were seen, occasionally extending into the superficial tumor aggregates. In the dermis, there were “lobulated nests” along with conspicuous melanocytic maturation. Given more than the occasional pagetoid scatter of individual melanocytes in the epidermis as well as proliferation between the rete ridges in some foci, the lesion was felt to represent a Spitz nevus with atypical features. Complete excision was recommended given margin involvement.

From the above-mentioned histological features [2], the tumor was suspected to harbor an *NTRK1* fusion, and this was confirmed through an RNA-based next generation sequencing assay, which detected a 5′ partner of *LMNA*. Although rare melanomas harboring *NTRK1* fusions exist, Spitz lineage more commonly portends indolent biologic behavior. In the unlikely event of disease progression, our patient would be a candidate for targeted therapy with TRK inhibitors.

## Conclusions

The presence of a dome-shaped red papule with a regular array of glomeruloid vessels admixed with crystalline structures should prompt clinical suspicion of a Spitz tumor with a *NTRK1* fusion. Although some variants of Bowen disease contain similar vessel morphology, the coexistence of crystalline structures and lack of scale would be unusual. Furthermore, the diffuse (as opposed to grouped) arrangement of the vessels is not typical for Bowen disease. Additional larger series are needed to replicate these findings and inform on the clinical-dermoscopic features of particular molecularly defined Spitz tumor subtypes.
